# Clinical presentation, management, and outcome of suspected central nervous system infections in Indonesia: a prospective cohort study

**DOI:** 10.1007/s15010-023-02170-0

**Published:** 2024-02-05

**Authors:** Kartika Maharani, Sofiati Dian, Ahmad Rizal Ganiem, Darma Imran, Riwanti Estiasari, Edwin Ardiansyah, Putri Widya Andini, Fransisca Kristina, David Pangeran, Lidya Chaidir, Bachti Alisjahbana, Andriansjah Rukmana, Ardiana Kusumaningrum, Robiatul Adawiyah, Decy Subekti, Evy Yunihastuti, Reyhan Eddy Yunus, Lia Waslia, Jakko van Ingen, Arjan van Laarhoven, Raph L. Hamers, Reinout van Crevel

**Affiliations:** 1 Department of Neurology, Faculty of Medicine, Dr. Cipto Mangunkusumo, General Hospital, Universitas Indonesia, Jakarta, Indonesia; 2 Department of Neurology, Faculty of Medicine, Dr. Hasan Sadikin General Hospital, Universitas Padjadjaran, Bandung, Indonesia; 3 Research Center for Care and Control of Infectious Disease (RC3ID), Faculty of Medicine, Universitas Padjadjaran, Bandung, Indonesia; 4 Department of Microbiology, Faculty of Medicine, Dr. Cipto Mangunkusumo General Hospital, Universitas Indonesia, Jakarta, Indonesia; 5 Department of Parasitology, Faculty of Medicine, Dr. Cipto Mangunkusumo General Hospital, Universitas Indonesia, Jakarta, Indonesia; 6 Oxford University Clinical Research Unit Indonesia, Faculty of Medicine, Universitas Indonesia, Jakarta, Indonesia; 7 Department of Internal Medicine, Faculty of Medicine, Dr. Cipto Mangunkusumo General Hospital, Universitas Indonesia, Jakarta, Indonesia; 8 Department of Radiology, Faculty of Medicine, Dr. Cipto Mangunkusumo General Hospital, Universitas Indonesia, Jakarta, Indonesia; 9 Department of Microbiology, Radboud Centre for Infectious Diseases (RCI), Radboud University Medical Centre, Nijmegen, The Netherlands; 10 Department of Internal Medicine, Radboud Centre for Infectious Diseases (RCI), Radboud University Medical Centre, Nijmegen, The Netherlands; 11 Center for Tropical Medicine and Global Health, Nuffield Department of Medicine, University of Oxford, Oxford, UK

**Keywords:** CNS infection, Indonesia, Adult, Diagnosis, Management, Outcome

## Abstract

**Background:**

Little is known about the etiology, clinical presentation, management, and outcome of central nervous system (CNS) infections in Indonesia, a country with a high burden of infectious diseases and a rising prevalence of HIV.

**Methods:**

We included adult patients with suspected CNS infections at two referral hospitals in a prospective cohort between April 2019 and December 2021. Clinical, laboratory, and radiological assessments were standardized. We recorded initial and final diagnoses, treatments, and outcomes during 6 months of follow-up.

**Results:**

Of 1051 patients screened, 793 were diagnosed with a CNS infection. Patients (median age 33 years, 62% male, 38% HIV-infected) presented a median of 14 days (IQR 7–30) after symptom onset, often with altered consciousness (63%), motor deficits (73%), and seizures (21%). Among HIV-uninfected patients, CNS tuberculosis (TB) was most common (60%), while viral (8%) and bacterial (4%) disease were uncommon. Among HIV-infected patients, cerebral toxoplasmosis (41%) was most common, followed by CNS TB (19%), neurosyphilis (15%), and cryptococcal meningitis (10%). A microbiologically confirmed diagnosis was achieved in 25% of cases, and initial diagnoses were revised in 46% of cases. In-hospital mortality was 30%, and at six months, 45% of patients had died, and 12% suffered from severe disability. Six-month mortality was associated with older age, HIV, and severe clinical, radiological and CSF markers at presentation.

**Conclusion:**

CNS infections in Indonesia are characterized by late presentation, severe disease, frequent HIV coinfection, low microbiological confirmation and high mortality. These findings highlight the need for earlier disease recognition, faster and more accurate diagnosis, and optimized treatment, coupled with wider efforts to improve the uptake of HIV services.

**Supplementary Information:**

The online version contains supplementary material available at 10.1007/s15010-023-02170-0.

## Introduction

Central nervous system (CNS) infections cause significant morbidity and mortality, especially in low-resource settings. The Global Burden of Disease study reported an estimated 2.82 million cases of meningitis globally with 318,400 deaths in 2016 [1]. CNS infections can present in several clinical syndromes, most frequently meningitis, followed by encephalitis, brain abscesses and myelitis [2, 3].

There are many barriers in the diagnosis and treatment of CNS infections: initial signs and symptoms are often nonspecific, leading to patient and doctor delays; physicians may be reluctant to perform diagnostic lumbar punctures (LPs); cerebral imaging, CSF analysis and specific microbiological tests are often unavailable, especially in resource-limited settings; and even with appropriate diagnostic modalities and appropriate treatment, morbidity and mortality remain high. Further context-specific research is needed to understand these barriers and take targeted actions to improve diagnosis, treatment and patient outcomes [4].

Indonesia, a lower-middle-income country with the world’s fourth largest population (275 million), continues to have a high burden of infectious diseases. Little is known regarding the etiology, management and outcomes of CNS infections and their association with the rising number of HIV cases in the country. Previous studies have been limited due to the relatively small number of participants being from a single center and/or focused on one specific pathogen [2, 5–9].

To address these knowledge gaps, we conducted a prospective cohort study in two large referral hospitals to examine the clinical presentation, etiology, treatment and outcome of patients with suspected CNS infections. As diagnosing CNS infections is difficult and timely treatment is vital, we also examined how clinical diagnosis evolved over time in these patients.

## Methods

### Study design and setting

We included adult patients with suspected CNS infections. Patients who refused to give consent were excluded. This prospective cohort study was conducted at two tertiary referral hospitals, Dr. Cipto Mangunkusumo Hospital in Jakarta and Hasan Sadikin Hospital in Bandung, West Java. Both hospitals serve patients in the two most populated provinces in Indonesia, with approximately 2000–3000 emergency department visits and 40,000–60,000 outpatient visits each month per hospital [10]. Patients are either referred from lower-level hospitals or primary health centers, and approximately two-thirds are self-referrals [11]. Both hospitals have neurologists with specific expertise on CNS infections and routine and microbiological CSF tests available, and both hospitals offer HIV services. Brain CT and MRI are available, although access may be constrained due to costs or limited capacity. Nearly all patients attending the hospitals are covered by the National Health Insurance scheme. Ethical approval was obtained from the institutional review board of Universitas Indonesia (No. 1365/UN.2.F1/ETIK/2018). Patients or their proxy provided written informed study consent, which included sample storage for future studies.

### Study procedures

We recruited consecutive patients ≥ 18 years of age who presented between April 2019 and December 2021 with suspected CNS infections, as judged by the attending physicians. Participants were recruited from the emergency room, neurology and HIV outpatient clinic, and medical and neurology wards. All participants underwent a standardized diagnostic work-up with systematic recording of signs and symptoms; neurological examination, including functional status using the modified Rankin scale [12]; and blood examination, including complete blood count (CBC), serum glucose, serum electrolytes, liver functions, creatinine, and HIV testing (followed by CD4 + T-cell count for those who tested positive). Routine CSF examination (cells, protein, glucose) was performed, except if LP was deemed contraindicated [13]. Clinical and ancillary data were documented in an electronic study database. We stored baseline CSF, blood samples, urine samples, and any isolated microbial pathogens for future study.

### Microbiological testing

A minimum of 10 mL cerebrospinal fluid (CSF) was collected unless contraindicated. Three milliliters was used for routine CSF analysis (leukocytes, protein, glucose), India ink, and Gram staining; bacterial culture was performed at the physician’s request. The remaining CSF (minimum 6 mL) was used for TB diagnostics, for which CSF was concentrated by centrifugation (3000 ×*g* for 15 min), and CSF sediment was used for microscopy, GeneXpert MTB/Rif, and mycobacteria growth indicator tube (MGIT or MODS) followed by Lowenstein-Jensen or Ogawa subculture. Cryptococcal antigen (CrAg) was measured in CSF from HIV-infected patients using a lateral flow assay (LFA).

In individual cases, attending physicians might decide on additional testing, including CSF real-time PCR for viral infection (herpes simplex virus, varicella-zoster virus, cytomegalovirus); fungal or bacterial culture; quantitative serum toxoplasma IgG for CNS toxoplasmosis; Rapid Plasma Reagin (RPR) and Treponema Pallidum Hemagglutination Assay (TPHA) for neurosyphilis; anti-NMDA receptor in suspected autoimmune encephalitis; and cytology assessment for CNS lymphoma.

### Radiological evaluation

Brain CT with contrast using a dual-source 128-slice (1.3 mm) scanner was part of routine procedures unless contraindicated. Brain MRI (1.5 T) with contrast was performed on clinical indication only. Neuroimaging assessment was performed systematically by the attending radiologists recording the presence or absence of abnormalities, including meningeal enhancement, hydrocephalus, tuberculoma, encephalitis or cerebritis, abscess, infarction, and herniation. Meningeal enhancement was defined as any pathological contrast enhancement of the meninges with linear or gyri form appearance [14]; hydrocephalus was defined as the distention of lateral cerebral ventricles with Evans ratio > 30% and/or the size of one or both temporal horns > 2 mm [15]; tuberculoma was defined as isohyperdense solitary or multiple lesions with ring, nodular, or irregular nonhomogeneous contrast enhancement [16]. Encephalitis or cerebritis refers to poorly marginated cortical or subcortical hypodensity with variable patterns of contrast enhancement [17]; abscesses as round, oval, or multiloculated space-occupying lesions with ring enhancement appearance [17]; infarctions as hypodense lesions on CT scan or variable intensity based on the age of infarction on MRI in a vascular distribution [15]; and herniation as parenchymal displacement to the adjacent structures due to the mass effect [18]. All participants underwent routine chest X-rays, with evaluation for signs of pulmonary TB [19].

### Evaluation of diagnostic process and treatment

Diagnoses were evaluated and reviewed by the study team at three time points. First, an ‘initial diagnosis’ was made < 24 h after initial evaluation by the neurologist and used to guide initial empirical treatment and further investigations. (2) A ‘discharge diagnosis’ was made at the time of hospital discharge or in-hospital death, updated by the study team according to further testing or clinical judgment. Possible treatment adjustments during hospitalization were recorded, and the discharge diagnosis was used to guide treatment and follow-up after discharge. (3) Finally, a ‘final diagnosis’ was made retrospectively by the clinical investigators taking into account all information available (e.g., including retrospective CSF testing). Throughout the process, as much as possible, standardized case definitions were followed (Table 1).

**Table 1:** Case definitions

Final Diagnosis	Definition		Refs.
CNS tuberculosis	TBM Definite	Positive CSF microscopy for acid-fast bacilli, *Mycobacterium tuberculosis* culture, or PCR (Xpert TB)	[20]
	TBM Probable	TBM research case definition score ≥ 10 (without available CT imaging) or ≥ 12 (with CT), and exclusion of alternative diagnoses	[20]
	TBM Possible	TBM research case definition score of 6–9 points (without available CT imaging) or 6–11 points (with CT), and exclusion of an alternative diagnosis	[20]
	Tuberculoma	Iso-hyperdense solitary or multiple lesions with ring, nodular, or irregular nonhomogenous contrast enhancement compatible with tuberculous etiology	[16]
Cerebral toxoplasmosis (presumptive)	HIV infection and presence of 1 or more cerebral mass lesions on CT or MRI or IgG antibody to *Toxoplasma*, and exclusion of an alternative diagnosis		[22]
Cryptococcal meningitis	Positive CSF India ink examination (budding encapsulated yeasts), or *Cryptococcus neoformans* culture, or cryptococcal antigen lateral flow antigen test		[23]
Viral encephalitis (CMV, HSV, VZV, or other viruses)	Definite	Positive CSF PCR for HSV, CMV, VZV, or other viruses with the present of clinical syndrome	[21]
	Probable	Clinical syndrome: Altered mental status plus 2 or more of the following: fever ≥ 38 °C; seizures; new onset of focal neurologic findings; CSF WBC count ≥ 5/mm3; abnormality of brain parenchyma on neuroimaging suggestive of encephalitis; abnormal EEG	
Bacterial meningitis	Definite	Meningitis with detection of bacteria in CSF by microscopy or culture	[40]
	Probable	Compatible clinical syndrome, positive blood culture, plus 1 of the following CSF changes: > 5 leukocytes/mm3; glucose < 40 mg/dL or CSF/blood glucose ratio < 0.5; or protein > 100 mg/dL	[40]
Bacterial brain abscess	Definite	Evidence of brain abscess seen during surgery or histopathological examination	[41]
	Radiologically confirmed	Brain CT and/or MRI with supporting evidence of brain abscess; and compatible clinical syndrome (headache, fever, localized neurological signs and/or impaired consciousness	
Neurosyphilis	Positive blood VDRL or TPHA and reactive CSF VDRL and/or elevated CSF cell count or elevated CSF protein or focal neurological signs plus exclusion of alternative diagnoses		[42]
CNS infection of unknown etiology	Suspected CNS infection based on combination of compatible clinical signs, CSF findings, and brain CT scan, and no other diagnosis established		Con-sensus

*TBM*  berculous meningitis, *CSF*  Cerebrospinal fluid, *PCR* Polymerase chain reaction, *MTB* *Mycobacterium tuberculosis*, *RIF* Rifampicin, *CT* Computed tomography, *MRI *Magnetic resonance imaging, *HSV* Herpes simplex virus, *VZV*  Varicella-zoster virus, *CMV*  Cytomegalovirus, *WBC*  White blood cells, *EEG * Electroencephalography, *RPR * Rapid plasma regain, *VDRL* venereal disease research laboratory, *CNS* Central nervous system

Initial treatment, based on clinical judgment and available baseline test results, was given in accordance with national/hospital guidelines (Supplement 1). Anti-retroviral (ART) initiation was performed according to the national guidelines 4–8 weeks after the start of therapy for TB meningitis, 2 weeks after the start of therapy for toxoplasma encephalitis, and 4–6 weeks after the start of therapy for cryptococcal meningitis. All patients with TB meningitis received adjunctive corticosteroids.

### Follow-up

We documented in-hospital and 6 month survival based on medical records and phone calls to patients and relatives. For patients who were alive at discharge or at 6 months, we recorded the Glasgow Coma Scale (GCS) and modified Rankin scale (mRS), a 6-point disability scale that measures dependence in performing daily activities [12]. Patients discharged from the hospital will receive monthly phone calls until the 6 month. Three unanswered calls will be considered lost to follow-up, and a social worker will conduct home visits to reach the patients. In the event of a patient's death during the follow-up period, the cause of death will be investigated using the WHO verbal autopsy method or by interviewing the family during the home visit.

### Statistical analysis

We made comparisons between groups according to HIV status using Chi-square/Fisher exact (categorical data) or parametric/nonparametric tests (numerical data). Survival analysis was presented using Kaplan‒Meier curves with log-rank tests, and univariate and multivariate Cox regression analyses were used to identify factors associated with death. All statistical analyses were performed using SPSS 23 for Windows (SPSS Inc., Chicago, IL). All figures were created with RStudio in R 4.4.2.

## Results

### Clinical characteristics and patient mortality

**Fig. 1:**
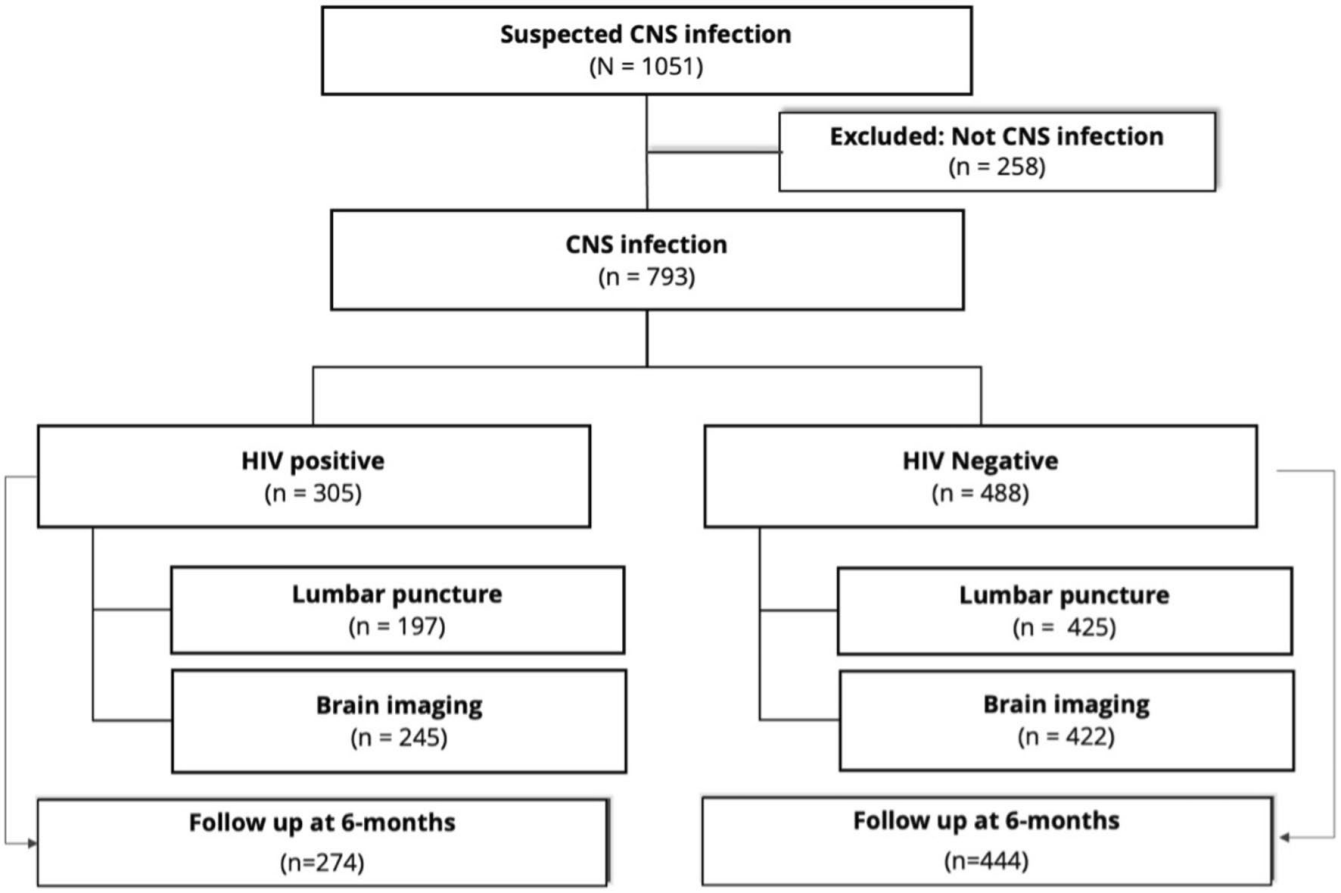
Patient flowchart

We screened 1051 patients with clinically suspected CNS infection for the study. We excluded 258 patients due to alternative diagnoses, such as primary headache, autoimmune-related neurological disorders, secondary syphilis, psychiatric disorders, or stroke. A total of 793 patients were enrolled, of whom 78% underwent lumbar puncture, and 84% had brain imaging (Fig. 1**)**. The median age was 33 (IQR 26–44) years, and the majority were male (62%) and self-referred (59%). The median time between the onset of neurological symptoms and hospital presentation was 14 days (IQR 7–30). The most frequent symptoms were fever, headache, seizures, lowered consciousness, motor abnormalities, and cranial nerve palsies (Table 2). HIV infection was present in 305 patients (38%), mostly at an advanced stage, with median CD4 T-cell counts of 22 (IQR 13–48) cells/mL among those newly diagnosed with HIV and 36 (IQR 12–120.5) cells/mL among those already known to have HIV. Brain imaging and lumbar puncture were performed a median of 24 and 26 h after admission, respectively. CSF examination and neuroimaging results are summarized in Table 2.

**Table 2:** Baseline characteristics (stratified by HIV status)

Variables	Data completeness	HIV positive (n = 305)	HIV negative (n = 488)	*p*
Age (years), median (IQR)	100%	36 (29.5–42)	32 (24–46)	0.02
Male, n (%)	100%	238 (78)	251 (51)	< *0.001*
HIV diagnosis and treatment				
New HIV diagnosis, n (%)	100%	43 (14)	-	
Known HIV, not on ART	95.4%	146 (59)	-	
Known HIV, history of recent ART	95.4%	102 (41)	-	
TB treatment, n(%)				
On admission	100%	42 (14)	83 (17)	0.1
History of previous TB treatment	100%	61 (20)	69 (14)	0.1
**Symptoms**				
Time since first neurological symptom (days), median (IQR)	98.1%	21 (7–60)	14 (6–30)	< *0.001*
Fever, n (%)	100%	151 (50)	339 (70)	< *0.001*
Headache, n (%)	100%	199 (65)	330 (68)	*0.5*
Loss of consciousness, n (%)	99.7%	159 (52)	344 (71)	< *0.001*
Seizures, n (%)	100%	55 (18)	113 (23)	*0.09*
Behavioral change, n (%)	97%	56 (19)	121 (26)	*0.02*
Vomiting, n (%)	100%	74 (23)	126 (25)	*0.7*
Chronic cough, n (%)	100%	72 (24)	144 (30)	*0.1*
**Signs**				
Body temperature > 38 (^o^C)	99.6%	43 (14)	78 (16)	0.5
GCS, median (IQR)	100%	14 (12–15)	13 (11–15)	< *0.001*
Neck stiffness, n (%)	98.6%	116 (38)	294 (61)	< *0.001*
Cranial nerve palsy, n (%)	99.7%	168 (55)	312 (64)	*0.01*
Motor abnormality, n (%)	100%	164 (54)	290 (59)	*0.1*
**Lumbar puncture & CSF analysis**				
Time to lumbar puncture (hours), median (IQR)	100%	29 (14–68)	24 (14–68)	0.5
Opening pressure (cmH_2_O), median (IQR)	31%	14 (8–20)	12 (10–17)	*0.8*
Leukocytes (cells/uL), median (IQR)	99.8%	7 (2–24)	32 (4–130)	< *0.001*
Neutrophils (%), median (IQR)	99.7%	18.5 (0–50)	24 (5–53.8)	*0.1*
Protein (mg/dL), median (IQR)	92%	56 (40–122.3)	125 (58–278)	< *0.001*
CSF glucose ratio, median (IQR)	93%	0.5 (0.4–0.6)	0.4 (0.2–0.6)	< *0.001*
**Blood**				
Moderate/severe anemia^a^, n (%)	99.2%	76 (25.4)	90 (18.4)	0.02
Leukocytes (× 10^9^/L), median (IQR)	99.1%	6.4 (4.5–9.4)	10.9 (7.7–14.6)	< *0.001*
Thrombocytes (× 10^9^/L), median (IQR)	99.1%	253 (184–318)	300 (214–386)	< *0.001*
Moderate/severe hyponatremia^b^, n (%)	96.7%	103 (36)	198 (41)	0.1
CD4 count (cells/mL), median (IQR)	62.9%	32 (12.3–98.8)	-	
**Brain imaging findings**				
Meningeal enhancement, n (%)	100%	59 (19.3)	190 (38.9)	< *0.001*
Infarct, n (%)	100%	41 (13.4)	76(16)	*0.4*
Hydrocephalus, n (%)	100%	34 (11)	115 (24)	< *0.001*
Tuberculoma, n (%)	100%	8 (2.6)	49 (10)	*0.001*
Herniation, n (%)	100%	26 (9)	8 (2)	< *0.001*
Brain abscess, n (%)	100%	30 (9.8)	26 (5.3)	*0.02*
Encephalitis, n (%)	100%	70 (23)	24 (4.9)	< *0.001*
**Mortality**				
At discharge	100%	82 (27)	152 (31)	0.2
At 6 months	90.5%	131 (48)	226 (51)	0.6

*ART*  antiretroviral therapy, *IQR*  interquartile range, *GCS*  Glasgow coma scale, *CSF*  cerebrospinal fluid

^a^Moderate/severe anemia defined as hemoglobin level < 10 mg/dL

^b^Moderate/severe hyponatremia defined as serum sodium level < 130 mEq/L

### Final diagnosis

Among HIV-uninfected patients (n = 488), CNS tuberculosis was the most common etiology (60%), while viral (8%) and bacterial disease (4%) were uncommon. Among HIV-infected patients (n = 305), cerebral toxoplasmosis was the most common diagnosis (41%), followed by CNS tuberculosis (19%), neurosyphilis (15%) and cryptococcal meningitis (10%; Table 3). Acute bacterial meningitis, bacterial abscess and viral encephalitis were uncommon in both groups. A microbiologically confirmed diagnosis was established in 198 of 793 patients with a suspected CNS infection (25%); a final diagnosis was reached by consensus or international guidelines in 58% [20–23]; and no clear etiology was established in 17% (Table 3). Among 325 patients diagnosed with TB meningitis, microbiological confirmation was reached in 154 (47%) patients by Xpert MTB/RIF (n = 58), culture (n = 45) and/or microscopy (n = 15).

**Table 3:** CNS infections, final diagnosis

No	Diagnosis	HIV status		Total (n = 793)
		HIV positive (n = 305)	HIV negative (n = 488)	
1	Tuberculosis	58 (19)	291(60)	349 (44)
	Definite TBM	15	139	154
	Probable TBM	40	131	171
	Tuberculoma	3	21	24
2	Cerebral toxoplasmosis	127 (41)	0 (0)	127 (16)
3	Neurosyphilis	46 (15)	3 (1)	49 (6)
4	Viral encephalitis	3 (1)	38 (8)	41 (5)
	Definite	0	5	5
	Clinically suspected	3	34	37
5	Cryptococcal meningitis	29 (10)	1 (0)	30 (4)
6	Brain abscess	2 (1)	23 (5)	25 (3)
	Definite	0	4	4
	Radiologically suspected	2	19	21
7	Bacterial meningitis	0 (0)	20 (4)	20 (3)
	Definite	0	5	5
	Clinically suspected	0	15	15
8	Other CNS infection^a^	2 (1)	13 (3)	15 (2)
9	Suspected CNS infection, unknown etiology	38 (12)	99 (20)	137 (17)

^a^Other infections included infectious myelitis, progressive multifocal leukoencephalopathy, cerebral aspergillosis, and subdural empyema

**Fig. 2:**
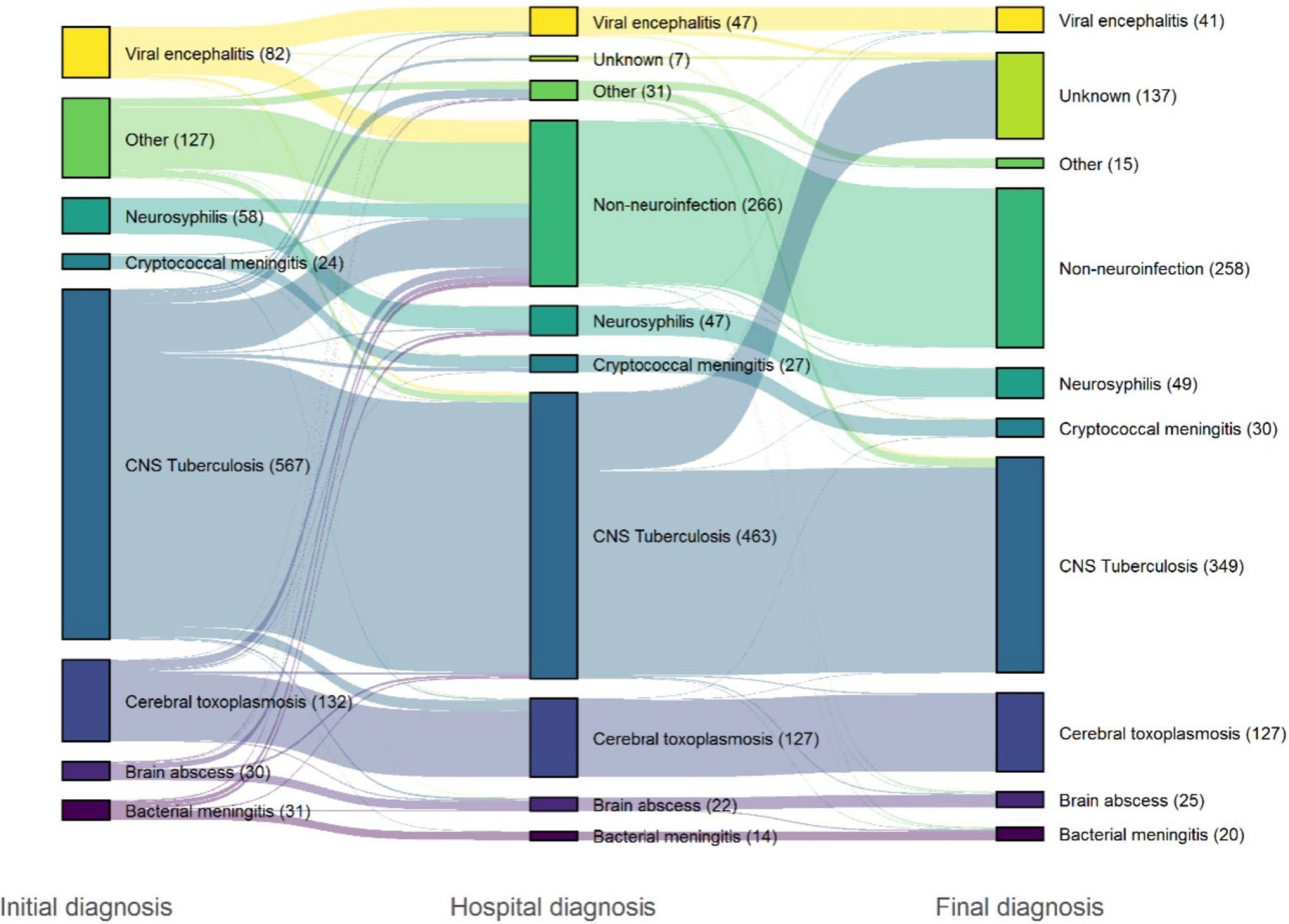
Patient diagnoses over time The initial diagnosis was recorded within 24 h of admission, the hospital diagnosis was determined after hospitalization, and the final diagnosis was determined after all test results were gathered. Each bar is connected by the same color ribbon to illustrate changes in diagnosis over time. The height of the bars represents the number of patients for each diagnosis.

The most common initial diagnosis was CNS tuberculosis (54%; Fig. 2). Of those who were initially diagnosed with CNS tuberculosis (n = 567), CNS infection was deemed unlikely at discharge in 79 (14%), and the final diagnosis was another CNS infection in 52 (9%). Conversely, in 18 patients (3%), CNS tuberculosis was not considered on admission but was diagnosed later during hospitalization with further test results and/or after exclusion of other diagnoses (Fig. 2). A correct initial diagnosis of cerebral toxoplasmosis, cryptococcal meningitis, and neurosyphilis was more likely based on cerebral imaging, serum IgG anti-toxoplasma, CSF cryptococcal antigen (CrAg) immunoassay test and VDRL, respectively. The median time between hospital presentation and appropriate treatment based on the initial diagnosis was 1 day (excluding those who were already on treatment for pulmonary TB when entering the cohort).

### Treatment outcome and prognostic factors

**Fig. 3a.**
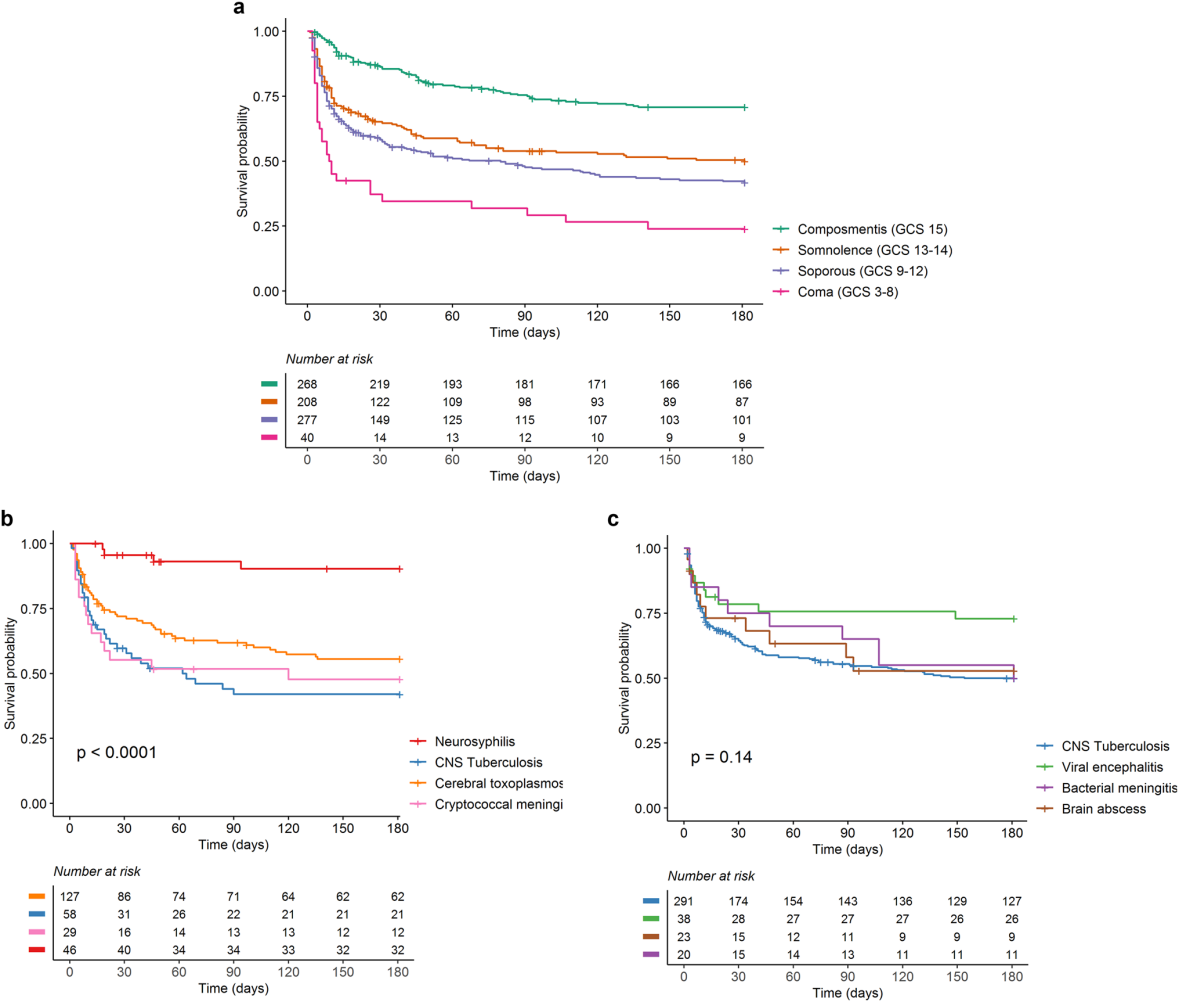
Patient mortality according to Glasgow coma scale at time of diagnosis **b**. Top 4 etiology in HIV positive. **c**. Top 4 etiology in HIV negative

The overall in-hospital mortality was 30% (234/793) and was highest for cryptococcal meningitis (47%, 14/30), followed by bacterial meningitis (35%, 7/20) and CNS TB (32%, 112/349). Among 721 (91%) participants for whom 6 month outcome data were available, 357 (50%) died, a median of 10 days after hospital admission. Mortality was higher and occurred earlier for those presenting with more severe neurological disease (Fig. 3a). Mortality appeared highest for those with unknown etiology, followed by TB meningitis in both groups (Fig. 3b, c). Among 137 patients with suspected CNS infection of unknown etiology, in-hospital and 6-month mortality was 35% and 52%, respectively. After exclusion of patients with neurosyphilis (all of whom survived), the risk of death was associated with older age, HIV infection, decreased consciousness at the time of admission, and other clinical (fever, headache), radiological (hydrocephalus) and CSF markers (pleocytosis and lower CSF glucose ratio) indicating severe disease (Table 4).

**Table 4:** Factors associated with 6 months mortality (n = 744)

Variable	*Univariate*		*Multivariate*	
	HR (95% CI)	*P*	HR (95% CI)	*P*
**Clinical**				
Age (per 10 year) increase	1.2 (1.1–1.3)	< 0.001	1.1 (1–1.3)	0.01
Male	1.0 (0.8–1.2)	0.7	0.7 (0.5–0.9)	0.04
HIV positive	1.1 (0.8–1.3)	0.5	1.8 (1.3–2.6)	0.002
**Symptoms**				
Time since first neurological symptoms (per 1 day increase)	1.0 (0.9–1.0)	0.8	1.0 (0.9–1.0)	0.4
Fever	1.5 (1.2–1.9)	< 0.001	1.5 (1.1–2.1)	0.02
Headache	0.9 (0.7–1.1)	0.4	0.9 (0.7–1.3)	0.6
Loss of consciousness	2.2 (1.7–2.9)	< 0.001	2.6 (1.7–3.9)	< 0.001
Seizure	0.9 (0.7–1.1)	0.3	0.9 (0.7–1.4)	0.8
Behavioral changes	0.9 (0.7–1.2)	0.6	1.0 (0.7–1.4)	0.9
Vomiting	0.9 (0.7–1.1)	0.4	0.8 (0.6–1.2)	0.3
Chronic cough	1.2 (0.9–1.5)	0.2	0.9 (0.6–1.2)	0.4
**Signs**				
Body temperature > 38 (^o^C)	1.4 (1.1–1.9)	0.01	1.2 (0.9–1.8)	0.3
GCS (per 1-point increase)	0.9 (0.9–0.9)	< 0.001	1.0 (1.0–1.1)	0.6
Neck stiffness	1.6 (1.3–2.0)	< 0.001	0.9 (0.6–1.3)	0.6
Cranial nerve palsy, n (%)	1.4 (1.1–1.7)	< 0.001	1.1 (0.8–1.6)	0.5
Motor abnormality(s), n (%)	1.4 (1.1–1.8)	< 0.001	1.3 (0.9–1.7)	0.04
**Lumbar puncture & CSF analysis**				
Opening pressure (per 1-point cmH_2_O increase)	1.0 (0.9–1.0)	0.3	NA	NA
Leukocytes (per 1 cells/µL increase)	1.0 (0.9–1.0)	0.2	1.0 (0.9–1.0)	0.04
Neutrophils (per 10% increase)	1.0 (0.9–1.0)	0.1	1.0 (0.9–1.0)	0.2
Protein (per 10 mg/dL increase)	1.0 (0.9–1.0)	0.3	1.0 (0.9–1.0)	0.9
CSF glucose ratio (per 0.10 increase)	0.4 (0.2–0.7)	< 0.001	0.3 (0.1–0.6)	0.001
**Blood**				
Moderate/severe anemia	1.5 (1.2–1.9)	< 0.001	1.1 (0.8–1.5)	0.7
Leukocytes (per 10^9^/L increase)	1.0 (0.9–1.0)	0.4	1.1 (1–1.02)	0.1
Thrombocytes (per 10^9^/L increase)	1.0 (0.9–1.0)	0.3	1.0 (0.9–1.0)	0.8
CD4 (per 10 cells/mL increase)	1.0 (0.9–1.0)	0.1	NA	NA
**Brain imaging findings**				
Meningeal enhancement	1.1 (0.9–1.3)	0.5	1 (0.7–1.3)	0.8
Infarct	1.1 (0.8–1.4)	0.7	1.1 (0.7–1.5)	0.8
Hydrocephalus	1.5 (1.2–1.9)	< 0.001	1.5 (1.1–2.1)	0.01
Tuberculoma	0.8 (0.5–1.2)	0.3	0.9 (0.5–1.6)	0.7
Herniation	0.8 (0.5–1.4)	0.5	1.2 (0.3–4.7)	0.8
Brain abscess	0.5 (0.3–0.9)	0.01	0.5 (0.2–1.5)	0.2
Encephalitis	0.8 (0.6 -1.2)	0.3	0.9 (0.6–1.5)	0.8

*GCS*  glasgow coma scale, *CSF*  cerebrospinal fluid, *NA*  Not applicable

**Fig. 4:**
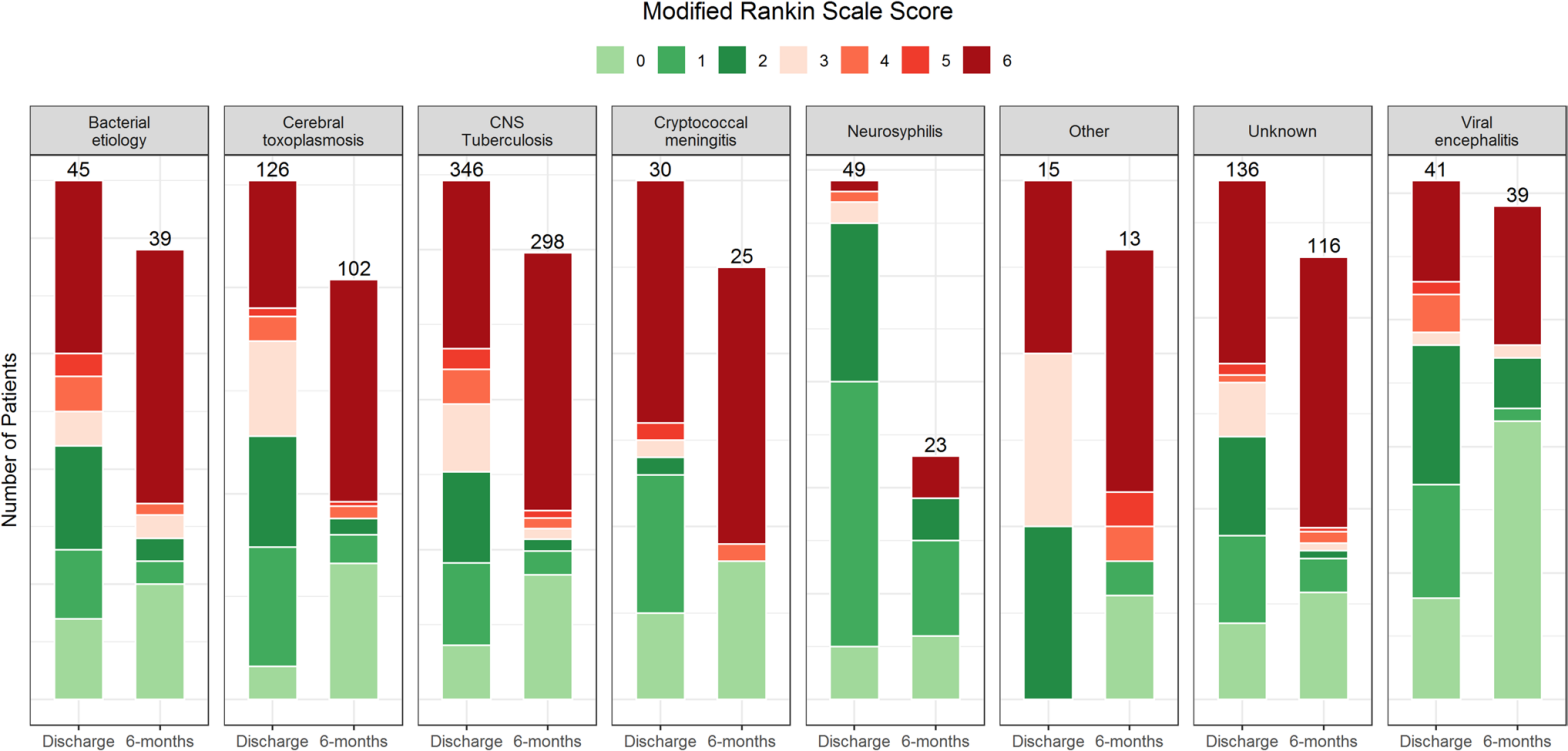
Functional outcome using the modified Rankin scale Functional outcome for those with available modified Rankin Scale data at discharge (99.4%) and after 6 months (82.6%), stratified by final diagnosis. MRS 0 = no symptoms; 1 = no significant disability; 2 = slight disability, 3 = moderate disability, 4 = moderately severe disability, 5 = severe disability, 6 = dead. The numbers above the bar refer to the total number of patients with available data at each time point.

Many patients who were alive after 6 months of follow-up suffered from long-term disabilities (Fig. 4). For instance, of 126 CNS tuberculosis patients (median age 29) with available MRS data at 6 months, 19 (15%) had moderate or severe disabilities and required support from others for their daily activities.

## Discussion

We prospectively evaluated all patients who presented with a suspected CNS infection over a 32-month period in two referral hospitals in Indonesia. Patients were mostly young and severely ill on presentation, and one-third suffered from advanced HIV infection. CNS tuberculosis was the most common final diagnosis overall, cerebral toxoplasmosis was the most common among HIV-infected patients, and viral and bacterial meningitis were uncommon. There were substantial diagnostic challenges, with only 25% of patients receiving a definite diagnosis. Establishing a timely diagnosis proved challenging, often leading to delays in the initiation of appropriate treatment. The overall in-hospital mortality was 30%, the six-month mortality was 45%, and significant disabilities were common among the patients who survived.

Patients in this cohort presented late and with very severe disease, similar to previous smaller studies from this setting [11], and often more severe than in studies from other countries. For instance, in neuro-infection cohorts in Vietnam [24] and England [3], 38% and 55% of patients (versus 66% in our cohort) were unconscious, and 4% and 36% had focal neurological deficits (versus 58% in our cohort). This difference might be attributed to challenges in timely access or referral to appropriate care. Despite being a tertiary-referral hospital, most patients were self-referred, and our group has established that patients with TB meningitis often visit many formal and informal health providers before receiving an appropriate diagnosis [25]. This underlines a need for interventions to increase early diagnosis and treatment, for instance, through enhancing health literacy among patients and health professionals in the community or facilitating timely referral to higher-level care.

Tuberculosis was the most common cause of CNS infection, similar to previous studies from Indonesia [11] and India [26], where tuberculosis accounted for 92/274 (34%) and 205/401 (51%) of cases, respectively. Obviously, this rate is much higher than that found in countries with a low tuberculosis burden, such as Australia (1.8% of 725 CNS infections) [2], Singapore (20% of 110 cases) [27], and Vietnam (14% of 617 cases) [28]. In those studies, viral and bacterial meningitis were more common, similar to studies from Africa, Europe, and the United States [29]. We hypothesize that many patients with acute CNS infections, such as those caused by *S. pneumoniae* or herpes simplex virus, may die at home and that those with self-resolving disease (such as those caused by entero- or arboviruses) might not reach our facilities either.

Establishing the etiology of CNS infections is difficult. Despite a thorough microbiological evaluation, microbiological confirmation was only made in 25% of cases. This is not unique for lower-resource settings. For instance, in a large CNS infection cohort from Australia and a meningitis cohort from the UK, 29% and 42% were of unknown etiology [2, 30]. Higher microbiological confirmation was reported from Singapore [27], Vietnam [28], and Thailand [31]; at least one pathogen was found in 60%, 52%, and 48% of cases, respectively. It is challenging to identify and diagnose patients with a rare but potentially life-threatening CNS infection in a timely manner amidst the multitude of patients presenting with nonspecific symptoms. Neuroimaging and CSF analysis may appear normal in early disease, and CSF testing is not sufficiently sensitive for many pathogens [32, 33]. In contrast to the difficulty of finding pathogens in the CNS, a report from a large cohort of acute febrile illnesses in Indonesia showed a positive laboratory finding in 67.5% of participants, mostly from blood samples [34]. Metagenomic next-generation sequencing (NGS) is now introduced as a promising tool to increase the diagnostic yield for CNS infections [35, 36].

Mortality in this study was high, and many survivors suffered from significant disabilities. Consistent with previous studies [37], HIV infection was strongly associated with poor outcome. This is likely due to the advanced stage of HIV in our cohort, with very low CD4 counts, both among those newly diagnosed with HIV and those already known with HIV (but either having dropped out of care or infected with resistant HIV viruses). This highlights the importance of earlier HIV testing and efforts to improve retention, adherence and viral load testing for patients on ART.

The strengths of the study are that it is a large cohort comprising all patients with suspected CNS infection (rather than with one particular infection, such as tuberculous or cryptococcal meningitis) recruited in two reference centers. Another strength is its comprehensive, largely standardized diagnostic workup and use of clear diagnostic classification. The study has several limitations. First, we were not able to apply MRI and the latest molecular tools to establish disease etiology, but this reflects the often-restricted diagnostic capabilities in hospitals in high-burden countries. Second, our findings may not be representative of other, smaller hospitals in Indonesia with different patient populations and etiologies. Both our hospitals have been engaged in clinical research related to TB meningitis and other CNS infections for years, and this has increased awareness and quality of patient management [23, 38, 39]. HIV testing, lumbar puncture rates, CSF analysis and treatment are even more challenging in smaller hospitals, and mortality might even be higher. Further research is therefore needed to help improve triage and referral, early diagnosis, appropriate treatment and better supportive care for patients with CNS infections in high-burden countries such as Indonesia.

### Supplementary Information

Below is the link to the electronic supplementary material.

Supplementary file1 (DOCX 16 KB) Patients with suspected or confirmed TBM received an intensive anti-TB fixed drug combination (FDC) containing rifampicin 150 mg/isoniazid 75 mg/pyrazinamide 400 mg/ethambutol 275 mg based on body weight and an additional dose of 300 mg rifampicin for 2 months, followed by a 10 month maintenance phase of rifampicin and isoniazid at the same dose. We followed the TB National Guideline to define the daily dose according to the patients’ body weight. The Bandung site also implemented a routine of high-dose rifampicin (20 mg/kg) for the initial 30 days of intensive treatment. Tapered-dose dexamethasone for 6–8 weeks was also given as the adjunctive treatment (reference). Suspected bacterial meningitis was treated with ceftriaxone IV 4 g/d for 2 weeks and dexamethasone 20 mg/d for 4 days. Acute viral encephalitis was given aciclovir 10 mg/kg/d for 2 weeks in suspected HSV as etiology or ganciclovir 5 mg/kg/12 h for 2 weeks in confirmed CMV. Probable cerebral toxoplasmosis was initiated with pyrimethamine 200 mg/d followed by 50–75 mg/d and clindamycin 2400 mg/d for 6 weeks. Definite cryptococcal meningitis received amphotericin-B IV 0.7-1 mg/kg/d and fluconazole 800 mg/d for 2 weeks. Fluconazole IV 1200 mg/d for 2 weeks was given as an alternative if amphotericin-B was not available. The neurosurgeon was consulted in case of a suspected bacterial brain abscess for abscess drainage, and this was empirically treated with Ceftriaxone IV 4 g/d and Metronidazole 1500 mg/d for 4 weeks (8 weeks if not surgically evacuated). In a suspected case of neurosyphilis, ceftriaxone IV 2 g/d for 2 weeks was administered. Treatment was then adjusted based on the definite diagnosis.

## Data Availability

Data will be made available upon personal contact to authors.
