# Supplementary material for: Clinical presentation, management, and outcome of suspected central nervous system infections in Indonesia: a prospective cohort study
Source: Infection. 2024 Feb 5;52(2):583–95. doi: 10.1007/s15010-023-02170-0 (PMC10954958; doi:10.1007/s15010-023-02170-0)
Supplement: Supplementary file 1 — Supplementary file1 (DOCX 16 KB) Patients with suspected or confirmed TBM received an intensive anti-TB fixed drug combination (FDC) containing rifampicin 150 mg/isoniazid 75 mg/pyrazinamide 400 mg/ethambutol 275 mg based on body weight and an additional dose of 300 mg rifampicin for 2 months, followed by a 10 month maintenance phase of rifampicin and isoniazid at the same dose. We followed the TB National Guideline to define the daily dose according to the patients’ body weight. The Bandung site also implemented a routine of high-dose rifampicin (20 mg/kg) for the initial 30 days of intensive treatment. Tapered-dose dexamethasone for 6–8 weeks was also given as the adjunctive treatment (reference). Suspected bacterial meningitis was treated with ceftriaxone IV 4 g/d for 2 weeks and dexamethasone 20 mg/d for 4 days. Acute viral encephalitis was given aciclovir 10 mg/kg/d for 2 weeks in suspected HSV as etiology or ganciclovir 5 mg/kg/12 h for 2 weeks in confirmed CMV. Probable cerebral toxoplasmosis was initiated with pyrimethamine 200 mg/d followed by 50–75 mg/d and clindamycin 2400 mg/d for 6 weeks. Definite cryptococcal meningitis received amphotericin-B IV 0.7-1 mg/kg/d and fluconazole 800 mg/d for 2 weeks. Fluconazole IV 1200 mg/d for 2 weeks was given as an alternative if amphotericin-B was not available. The neurosurgeon was consulted in case of a suspected bacterial brain abscess for abscess drainage, and this was empirically treated with Ceftriaxone IV 4 g/d and Metronidazole 1500 mg/d for 4 weeks (8 weeks if not surgically evacuated). In a suspected case of neurosyphilis, ceftriaxone IV 2 g/d for 2 weeks was administered. Treatment was then adjusted based on the definite diagnosis. [file 15010_2023_2170_MOESM1_ESM.docx]

**Supplement 1: treatment regimens**

Patients with suspected or confirmed TBM received intensive anti TB fixed drug combination (FDC) containing rifampicin 150 mg/isoniazid 75 mg/pyrazinamide 400 mg/ethambutol 275 mg based on body weights; and additional dose of 300 mg rifampicin) for 2 months, followed by 10-month maintenance phase of Rifampicin and Isoniazid with the same dose. We followed the TB National Guideline to define the daily dose according to the patients’ body weight. (Bandung?). Tapered dose dexamethasone for 6-8 weeks was also given as the adjunctive treatment (reference).

Suspected bacterial meningitis was treated with ceftriaxone IV 4 g/d for 2 weeks and dexamethasone 20 mg/d for 4 days. Acute viral encephalitis was given aciclovir 10 mg/kg/d for 2 weeks in suspected HSV as aetiology, or ganciclovir 5 mg/kg/12 hours for 2 weeks in confirmed CMV. Probable cerebral toxoplasmosis was initiated pyrimethamine 200 mg/d followed by 50-75 mg/d and clindamycin 2400 mg/d for 6 weeks. Definite cryptococcal meningitis received amphotericin-B IV 0.7-1 mg/kg/d and fluconazole 800 mg/d for 2 weeks. Fluconazole IV 1200 mg/d for 2 weeks was given as an alternative if amphotericin-B was not available. The neurosurgeon was consulted in case of a suspected bacterial brain abscess for abscess drainage and this was empirically treated with Ceftriaxone IV 4 g/d and Metronidazole 1500 mg/d for 4 weeks (8 weeks if not surgically evacuated). In a suspected case of neurosyphilis, ceftriaxone IV 2 g/d for 2 weeks was administered. Treatment was then adjusted based on the definite diagnosis.
